# Radiation Therapy for Primary Cutaneous Rosai-Dorfman Disease of the Face: A Case Report Resistant to Multiple Lines of Treatment and a Literature Review

**DOI:** 10.7759/cureus.88864

**Published:** 2025-07-27

**Authors:** Omar G Lopez Navarro, Eduardo E Cervera-Ceballos, Adela Poitevin-Chacón, Hidralba Perez-Lopez, Fabiola Flores-Vazquez, Víctor I Urbalejo-Ceniceros, Jose A Arriaga-Marroquin, Alejandro Aviles-Salas, Irving E Sanchez-Rodriguez, Alejandro Villalvazo, Enrique Gutierrez-Valencia, Tomas F Gonzalez-Espinosa

**Affiliations:** 1 Hematology and Medical Oncology, Medica Sur, Mexico City, MEX; 2 Hematology, Instituto Nacional de Cancerología, Mexico City, MEX; 3 Radiation Oncology, Medica Sur, Mexico City, MEX; 4 Radiation Oncology, American British Cowdray Medical Center, Mexico City, MEX; 5 Hematology, National Cancer Institute of Mexico, Mexico City, MEX; 6 Pathology, National Cancer Institute of Mexico, Mexico City, MEX; 7 Radiation Oncology, Mexican Institute of Social Security, Guadalajara, MEX; 8 Radiation Oncology, Princess Margaret Cancer Centre, University of Toronto, Toronto, CAN

**Keywords:** chemotherapy, extranodal sinus histiocytosis with massive lymphadenopathy, primary cutaneous rosai-dorfman disease, radiation therapy, rare case report

## Abstract

Rosai-Dorfman disease (RDD) is a rare subtype of sinus histiocytosis, with primary cutaneous RDD (PCRDD) being an even rarer form, characterized by cutaneous lesions without systemic involvement. PCRDD presents a significant diagnostic challenge and is often resistant to conventional therapies, including surgical resection, steroids, and chemotherapy. Currently, no established management guidelines exist for this condition. Radiation therapy is emerging as a promising treatment option, especially for refractory cases involving cosmetically sensitive or difficult-to-resect areas. This case report presents a patient with treatment-resistant PCRDD affecting the face, which was refractory to multiple therapeutic approaches, including chemotherapy. The patient achieved durable remission with radiation therapy at an 18-month follow-up, highlighting the efficacy of this modality in managing resistant PCRDD and the importance of a multidisciplinary approach.

## Introduction

Rosai-Dorfman disease (RDD) is a subtype of sinus histiocytosis, also called systemic RDD. The disease course in both children and young adults is characterized by the development of non-painful bilateral lymphadenopathy [1]. Primary cutaneous RDD (PCRDD) is defined as the presence of cutaneous lesions caused by histiocytic proliferation in the absence of lymphadenopathy and/or extracutaneous disease [2]. PCRDD makes up 3% of cases of systemic RDD [3,4]. Here, we report a case of PCRDD that proved refractory to multiple lines of therapy, including chemotherapy. Remarkably, radiation therapy led to a complete response of the lesions, highlighting its potential as an effective option in the management of treatment-resistant PCRDD.

## Case presentation

**Figure 1:**
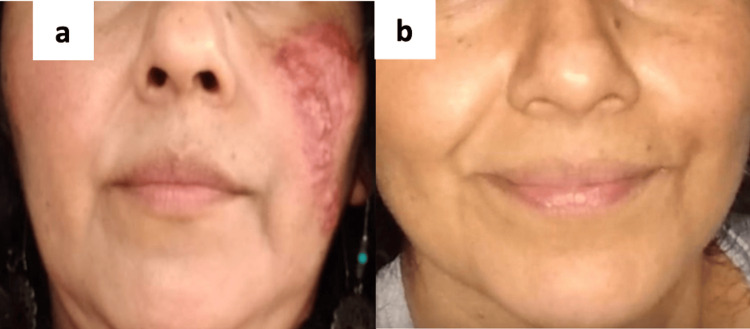
Malar and cheek regions before and after radiation therapy. a) First medical visit to the dermatology department. The patient presented with erythematous, red-brown, and red-yellow papules, comedones, and nodules on both malar and cheek regions. b) Follow-up visit after one year of completion of radiation therapy, showing complete resolution of the lesions.

A 47-year-old woman with a medical history of penicillin allergy and herpes virus infection in her right ear, but without other significant comorbidities, presented with a two-year history of erythematous, red-brown and red-yellow papules, nodules, and comedones on both cheeks, predominantly the left one (Figure 1a).

**Figure 2:**
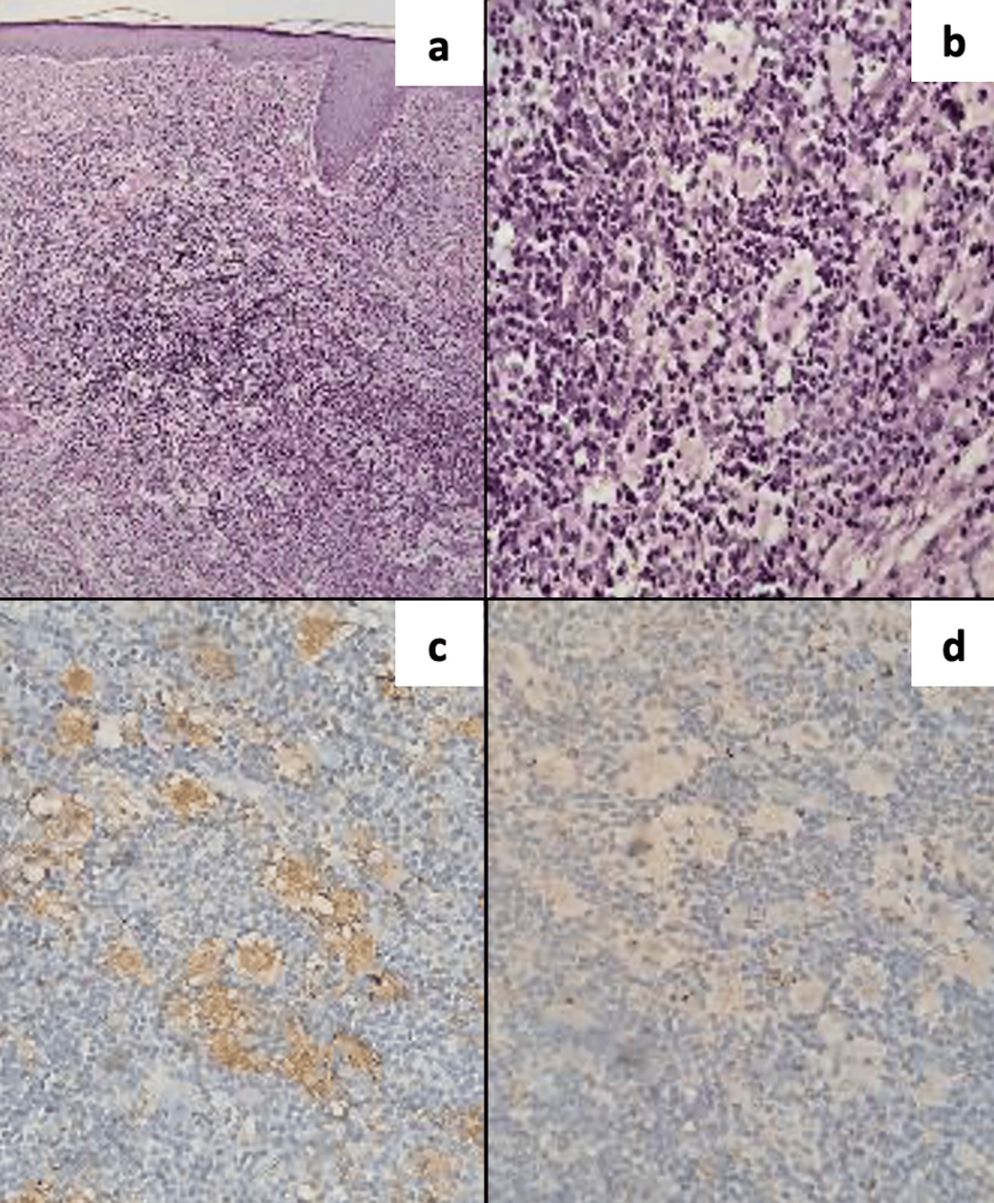
The biopsy of the lesion demonstrates involvement of all dermal layers (superficial, middle, and deep), showing histiocytes, emperipolesis, plasma cells with Russell bodies, lymphocytes, neutrophils, hemorrhage, fibrosis, and the expression of S-100 and CD68 proteins. a) 10× power field view. b) 40× power field view. c) Positive staining for S-100. d) Positive staining for CD68.

The patient underwent several dermatological treatments with no response. During her last visit to a dermatologist, she denied fever, malaise, diaphoresis, or other symptoms related to being immunocompromised. The physical examination was unremarkable. A biopsy from lesions of the left cheek indicated that all layers of the dermis (superficial, middle, and deep) featured histocytes, emperipolesis; a pathognomonic feature, plasma cells with Russell bodies, lymphocytes, neutrophils, hemorrhage, and fibrosis; the tissue also tested positive for the S-100 and CD68 proteins and negative for CD1A (Figure 2).

**Figure 3:**
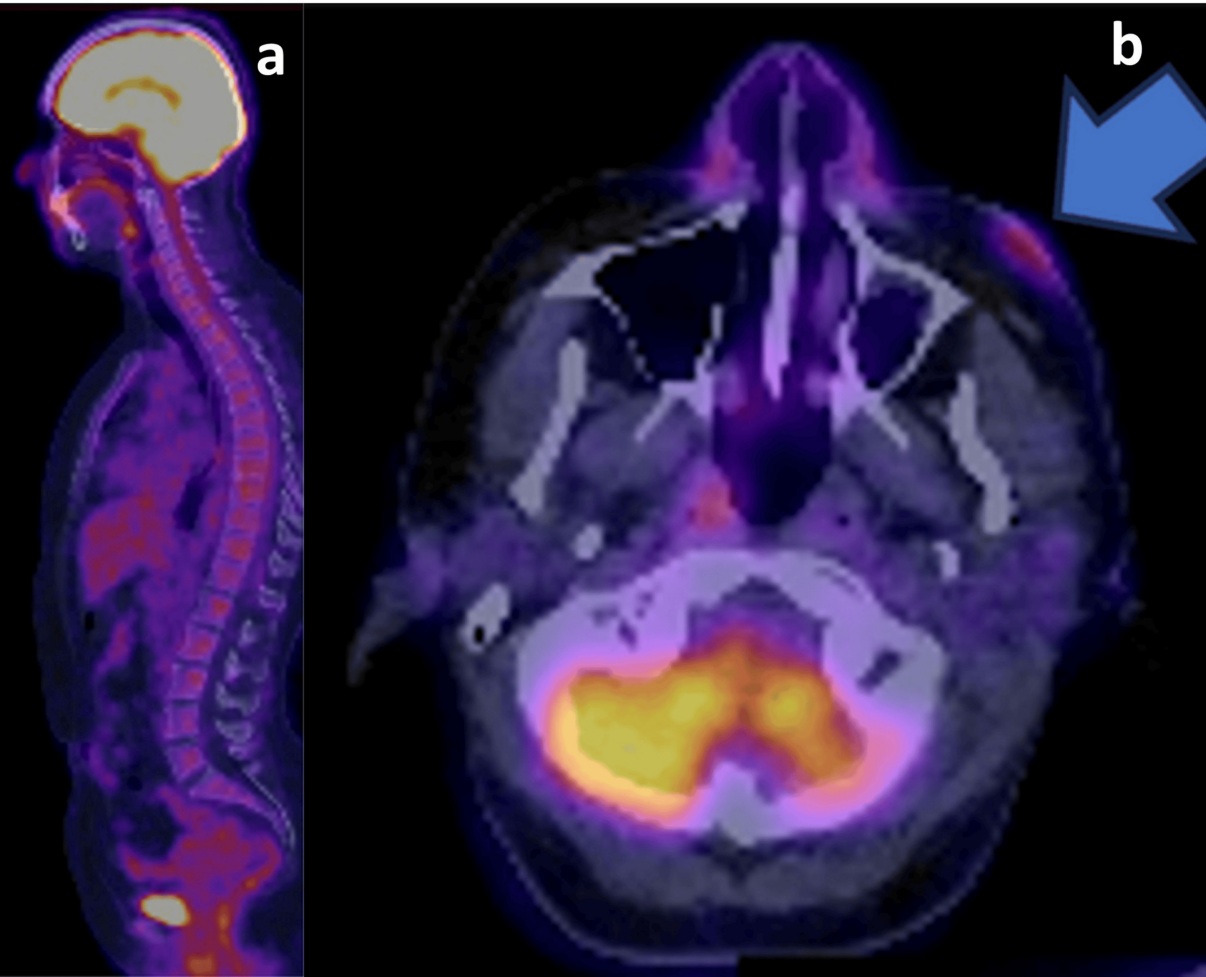
a) Sagittal view of FDG-PET/CT. b) Avidity of the soft tissue of the malar region (blue arrow). FDG-PET/CT: fluorodeoxyglucose positron emission tomography/computed tomography

In a laboratory work-up that included a complete blood count, measurement of antinuclear antibodies, and a metabolic panel, all results were normal. A fluorodeoxyglucose positron emission tomography/computed tomography (FDG-PET/CT) scan demonstrated bilateral avidity of the soft tissue in the malar region, with thickening of the left side and a maximum standardized uptake value (SUVmax) of 4.5 (Figure 3).

Cosmetic concerns made surgical excision of the affected facial area unfeasible. The patient was initially treated with topical and intralesional steroids, but the disease persisted. Second-line treatment with methotrexate resulted in stable disease, followed by six cycles of cyclophosphamide, which achieved a partial response. However, the disease progressed, prompting a switch to rituximab, purinethol, and dexamethasone for three cycles, again with only a partial response. Three weeks after the last rituximab cycle, the number and size of nodules and papules increased. Given the chemotherapy-resistant nature of the patient’s PCRDD, our institutional tumor board recommended definitive radiation therapy. The patient underwent localized electron beam radiotherapy using a bolus and bilateral ocular shielding. Each malar lesion was targeted with a 5 mm margin. A total dose of 48 Gy was delivered to the larger lesions in the left malar region, and 44 Gy to the smaller lesions in the right malar region, both administered in daily fractions of 2 Gy. After radiation therapy, the patient developed mucositis grades 1-2 and radioepitelitis grade 1. Radiotherapy was completed in April 2023, with a good response observed in the lesions, as evidenced by a decrease in plaque size, but persistent erythema remained. Then the patient continued maintenance therapy with purinethol on Fridays and methotrexate on weekends for one year, eventually achieving complete resolution of the lesions (Figure 1b).

## Discussion

RDD was first described by Destombes in 1965 and later characterized as a distinct clinicopathological entity by Juan Rosai and Ronald Dorfman in 1969, who reported four cases of massive cervical lymphadenopathy with hallmark histopathological features, coining the term “sinus histiocytosis with massive lymphadenopathy” [5,6]. Classic RDD predominantly affects younger individuals, with a male-to-female ratio of approximately 1.4:1, and is more frequently observed in African American and Caucasian populations than in Asians [1]. In contrast, PCRDD tends to present in older adults (mean age 43.5 years), shows a clear female predominance (up to 2:1), and is most often reported in Asian and Caucasian patients, suggesting important epidemiological and clinical distinctions between systemic and cutaneous forms of the disease [7]. RDD typically presents with bilateral, painless lymphadenopathy but may also include fever, night sweats, weight loss, leukocytosis, neutrophilia, polyclonal hyperglobulinemia, an elevated erythrocyte sedimentation rate, and immunological dysfunction. Clinical manifestations vary, ranging from asymptomatic skin nodules to end-organ dysfunction. Unfavorable or fatal outcomes are uncommon, but deaths have been reported in cases with multiorgan involvement [7,8]. Extranodal presentations have been reported in 30-75% of cases, including in the brain, head and neck region, nose, paranasal sinuses, thyroid, breast, bone, soft tissue, heart, gastrointestinal tract, kidney, and testis. Recently, RDD has been documented with craniospinal, intraparenchymal, intramedullary, and leptomeningeal involvement [1,4,8-10]. The skin is the most frequently affected extranodal site in RDD. [1] PCRDD has been observed in healthy patients with normal laboratory tests [4,8-10]. This condition was first described in 1978 in a 48-year-old male with a solitary shoulder nodule [11].

Pathology

The pathological features of PCRDD include polymorphic inflammatory infiltrates with small lymphocytes, plasma cells, and large histiocytes characterized by open chromatin in the nucleus, a single prominent nucleolus, and pericapsular fibrosis [12]. A pathological hallmark is emperipolesis or nondestructive phagocytosis; however, this may also be observed in idiopathic myelofibrosis, autoimmune hepatitis, thymoma, Hodgkin lymphoma, and autoimmune lymphoproliferative syndrome [13,14]. An important histopathological feature of PCRDD is an abundance of plasma cells, inflammatory infiltrates (histiocytes, lymphocytes, and neutrophils), fibrosis, a histiocytic immunophenotype, emperipolesis, the expression of the S100 and CD68 proteins, and the lack of CD1a expression [4,14].

Molecular biology

Molecular studies have reported that approximately one-third of RDD patients harbor mutations in genes involved in the mitogen-activated protein kinase/extracellular-signal-regulated kinase (MAPK/ERK) signaling pathway, most commonly NRAS, KRAS, and MAP2K1, and more rarely BRAF, supporting a neoplastic rather than reactive etiology [15-17]. These findings open the possibility for targeted therapy in selected cases, particularly using MEK inhibitors, such as trametinib and cobimetinib, in patients with MAP2K1 or other pathway-related mutations. Although clinical data are still limited, early reports suggest potential benefit, especially in systemic or refractory disease. Further research is needed to validate these therapeutic strategies and to determine the role of routine molecular profiling in guiding individualized treatment for RDD [9,17,18].

Risk factors

The etiology of RDD remains unknown. Viral and autoimmune causes have been suggested. Human herpesvirus 6 (HHV-6) DNA and associated antigens have been detected in lesions of patients with systemic RDD, although there is only one report of primary cutaneous cases [4]. IgG4-positive plasma cells have been observed in all RDD cases, along with a significantly increased IgG4/IgG ratio, which may suggest a link to IgG4-related sclerosing diseases [4,19]. However, the relationship between IgG4+ plasma cells and S-100+ Rosai-Dorfman histiocytes in PCRDD remains unclear [4].

Clinical features of PCRDD

Cutaneous skin lesions may be composed of papules; red, yellow, purple, or brown discoloration; solitary nodules, in groups or scattered; and erythema or hyperpigmentation. Lesions can be present anywhere on the body, but the extremities are the most frequent site, followed by the trunk and face [1]. A review that included one of the largest series of PCRDD patients reported the clinical morphology of this condition and proposed three main types of skin lesions for better recognition and understanding: papulonodular, indurated plaque, and tumor type, as mentioned by Kong et al. (Table 1) [1].

**Table 1:** Main types of lesions in primary cutaneous Rosai-Dorfman disease. Original table featuring the proposed classification by Kong et al. [1].

	Incidence	Characteristics	Size
Papulonodular type	79.5%	Clustering or satellite, papules and/or nodules with red, purple, or brown discoloration, papules and nodules could coalesce into dark red to violaceous plaques with nodules or papules, with an irregular surface	From 2 × 3 cm to 8 × 12 cm
Indurated plaque type	12.8%	Usually presented as a flat hyperpigmented plaque with a palpable infiltrated edge	Up to 10 cm
Tumor type	7.7%	Pink, dome-shaped, exophytic large mass, surrounded by a few papules; central ulceration may be present	From 3 cm to 5.5 cm

Diagnosis

The diagnosis is based on a combination of clinical features and histopathological findings, including the classification of lesions (Table 1), the presence of polygonal histiocytes with emperipolesis, positive staining for S-100 and CD68 proteins, negative staining for CD1a, and the absence of phlebitis [1,4]. Among these, the most critical features are the identification of characteristic polygonal histiocytes showing emperipolesis and positive immunoreactivity for S-100 [1]. These findings also rule out key differential diagnoses such as Langerhans cell histiocytosis (LCH) and lymphoma. In particular, the lack of CD1a expression excluded LCH, while the absence of clonality, along with the mixed inflammatory background and absence of atypical lymphoid populations, did not support a diagnosis of lymphoma.

Current trends in management and future directions

Due to its generally benign course and frequent spontaneous remission, PCRDD rarely causes mortality, and treatment should aim to minimize morbidity. A multidisciplinary approach integrating dermatology, oncology, radiation oncology, and hematology is key to ensuring accurate diagnosis and selecting the most appropriate, individualized therapy, especially in complex or refractory cases as ours. Expectant management is recommended when feasible. CRDD has demonstrated partial or complete responses to treatment in 16.1% of cases, with a mean remission time of 10 months, regardless of the treatment administered [20]. CRDD is a difficult entity to treat, showing a poor response to all current therapies; according to one report, the cure rate globally is 28.6% considering all therapeutic options [20]. The current treatment options include topical, systemic, and intralesional steroids; systemic treatments such as methotrexate, mercaptopurine, etoposide, cyclosporine A, dapsone, thalidomide, isotretinoin, and imatinib; and local therapies such as radiation and surgery [21-24]. Surgery is the most effective treatment, especially for small, isolated lesions in non-cosmetically sensitive areas. In contrast, corticosteroids are generally the least effective, with limited and shorter responses [20].

Patients with CRDD can receive active treatment for lesions that affect cosmetic appearance or that cause pain or itching. Localized radiation therapy is an effective therapeutic option for patients with PCRDD or CRDD, particularly in areas of high morbidity that are challenging to resect or that are cosmetically sensitive, such as the face [24]. There are no specific treatment guidelines for RDD, CRDD, or PCRDD. Reported doses of radiation therapy have been extrapolated from cutaneous lymphoma guidelines, ranging from 20 to 40 Gy and up to 50 Gy [25,26]. Our dose selection (44 Gy and 48 Gy in 2 Gy fractions) was based on clinical judgment, the extent of the lesions, and previous reports describing successful outcomes with doses ranging from 20 to 50 Gy [25,26]. While lower doses may be sufficient in select cases, we opted not to de-escalate treatment due to the refractory nature of the disease, lesion size, and their location in the facial area. Further studies are needed to evaluate the minimum effective dose and determine whether de-escalation may be appropriate in non-refractory or smaller lesions. The natural course of PCRDD varies. Without treatment, it may follow an indolent path, show spontaneous regression, or slowly progress. After local therapy, the disease may achieve prolonged remission. PCRDD has been reported to have an average duration of 19 months [2]. The present patient has had facial skin lesions for more than two years. The prognosis of PCRRD is good, with a low risk of developing systemic disease [1].

Radiation therapy can be a useful treatment for resistant CRDD and may be indicated for regions that can be challenging to resect or would have poor cosmetic outcomes. A delay in the diagnosis can be problematic cosmetically and negatively affect the quality of life; most patients are middle-aged women, like our case. Indeed, for all these reasons, our patient wanted active treatment. A search of the literature of other case reports of PCRDD besides our case was performed (Table 2) [2-4,21,22,24,27-41]. One study reported 77 cases localized to the face [2]. We identified five other similar cases [2,33-35,39,40]. A few cases have shown an aggressive clinical course, refractory or recurrent to therapy, requiring systemic therapy and/or local therapy.

**Table 2:** Comparison of our clinical case to the literature. References: [1,2-4,21,22,24,26-41]

Characteristics	Literature	Present case
Age (mean)	15-68 (43.5)	47
Sex M:F	1:1.8	F
Race	Asian and Caucasian	Hispanic
Clinical presentation	Cutaneous lesions with papules, nodules, with brown, purple discoloration, erythema, or hyperpigmentation	Malar plaque lesions, nodulopapular red-brown comedones on both cheeks, with predominance on the left cheek
Laboratory work up	Normal	Normal
Histological features	Emperipolesis, fibrosis, inflammatory infiltrates (plasma cells, histiocytes, lymphocytes, and neutrophils)	Superficial, middle and deep dermis with histocytes, with emperipolesis, plasma cell with Russell bodies (eosinophilic intracytoplasmic globules), lymphocytes, neutrophils, hemorrhagic, and fibrosis
Immunophenotype	S100+, CD68+, CD1A-	S100+, CD68+, CD1A-

Few cases of CRDD have been treated with radiotherapy, with variable efficacy [3,24,30,42,43]. To the best of our knowledge, only three cases of PCRDD (involving the face, nose, and ear) have been treated with radiation therapy (Table 3) [3,42,43]. Our patient is the fourth such case (involving the face). Like our case, there have been reports of recurrent, resistant CRDD that did not respond to steroids and required eight cycles of polychemotherapy (with rituximab, cyclophosphamide, vincristine, and prednisolone), followed by radiation [30,43].

**Table 3:** Comparison of clinical data of the present case with other reported cases of patients treated with radiation therapy to the face. References: [3,42,43]

Author/year of publication	Sex/age/race or nationality	Clinical presentation	Location/size	Time of evolution of lesion	Radiation dose/treatment technique	Toxicity	Follow-up/response	Adjuvant/maintenance therapy
Bunick et al., 2012 [3]	Male/37/Caucasian	Red, tender, nodular, nonscaly plaque	Right ear, superior helix, sparing the ear lobule	Three to six months	3000 cGy/15 fractions with intensity-modulated radiation therapy (IMRT)	None	Improved cosmetic appearance/10 months of follow-up, complete response	No
Annessi et al., 1996 [42]	Female/38/Italian	Asymptomatic	Posterior part of legs, nodular lesion of the nose	Two weeks	14 Gy in seven fractions in seven weeks/with kV Roentgen therapy	No side effects observed	Three years/complete response	No
Maklad et al., 2013 [43]	Male/26/Saudi	Erythematous lesion	Right cheek, left upper eyelid/ 3 × 3 cm and 1 × 2 cm	Six months	30 Gy/15 fractions with IMRT	None reported	One year/partial response, reirradiation needed	Rituximab, cyclophosphamide, vincristine, and prednisone
Present case	Female/47/Mexican	Erythematous, malar red-brown and red-yellow papules, nodules, and comedones	Skin of cheeks, predominant in left cheek	Two years	48 Gy and 44 Gy in 2 Gy fractions, left and right malar and cheek regions using 6 MeV electron beam therapy.	Mucositis grades 1-2 and radioepitelitis grade 1	More than one year/complete response	Purinethol on Fridays and methotrexate on weekends for one year

## Conclusions

PCRDD is a rare entity for which expectant management is recommended when feasible. There is no universally effective treatment, although surgery may yield favorable outcomes in select cases. Patients with PCRDD may benefit from active treatment when lesions are painful, pruritic, or cosmetically distressing. A multidisciplinary approach is strongly advised. CRDD and PCRDD should not be confused with other benign or malignant cutaneous conditions, as early detection and accurate diagnosis are crucial to avoid unnecessary morbidity from overly aggressive therapies. While local radiation therapy appears to be an effective option for lesions in cosmetically sensitive or surgically challenging areas, as demonstrated in the present case, there are no established dose guidelines for PCRDD, with reported ranges from 20 to 50 Gy in the literature. Previous reports and clinical judgment guided our radiation regimen using electron beam therapy (44 and 48 Gy in 2 Gy fractions). The role of maintenance therapy in long-term disease control remains unclear, and our follow-up is limited by its relatively short duration after tapering systemic treatment. Further studies are needed to determine optimal dosing, clarify the role of adjuvant therapy, and improve understanding of PCRDD pathophysiology to inform future therapeutic strategies.
